# Requirement of a putative mitochondrial GTPase, GemA, for azole susceptibility, virulence, and cell wall integrity in *Aspergillus fumigatus*

**DOI:** 10.3389/fmicb.2022.957857

**Published:** 2022-08-09

**Authors:** Xiaogang Zhou, Guorong Yang, Chengxi Li, Fan Yang, Xuelian Chang

**Affiliations:** Anhui Key Laboratory of Infection and Immunity, School of Basic Medicine, Bengbu Medical College, Bengbu, China

**Keywords:** *Aspergillus fumigatus*, mitochondria, drug resistance, GemA, cell wall

## Abstract

Drug resistance in fungal pathogens is a new challenge in clinical aspergillosis treatment. Mitochondria as dynamic organelles are involved in numerous biological processes in fungi, including drug resistance. However, little is known about the mechanism underlying mitochondrial regulation of the response of fungal pathogens to antifungal drugs. Here, we showed that a putative mitochondrial GTPase, GemA, a yeast Gem1 homolog, is crucial for the azole response and cell wall integrity in the opportunistic pathogen *Aspergillus fumigatus*. The fluorescence observation showed that GFP-labeled GemA is located in mitochondria, and loss of *gemA* results in aberrant giant mitochondrial morphology and abnormal mitochondrial membrane potential. Moreover, a Δ*gemA* mutant attenuates fungal virulence in the *Galleria mellonella* model of aspergillosis. Furthermore, *gemA* loss increases resistance to azoles and terbinafine but not to amphotericin B. Of note, RNA-seq combined with RT-qPCR showed that a series of drug efflux pumps were upregulated in the *gemA* deletion mutant. Deleting *mdr1* or inhibiting the expression of drug efflux pumps can partially decrease the resistance to azoles resulting from the *gemA* mutant, implying that GemA influences azole response by affecting the expression of drug efflux pumps. Importantly, the Δ*gemA* mutant is susceptible to the cell wall-perturbing reagent CR, but not to CFW, and this defect can be partly rescued by hyperosmotic stress. TEM revealed that the cell wall of Δ*gemA* was thicker than that of the WT strain, demonstrating that GemA plays a role in cell wall composition and integrity. Collectively, we identified a putative mitochondrial GTPase, GemA, which is critical for hyphal growth, virulence, azole susceptibility, and cell wall integrity and acts by affecting mitochondrial function.

## Introduction

Invasive aspergillosis is a fungal disease with high morbidity and mortality in immunocompromised patients (Hope, 2009; Bertuzzi et al., 2014; Arias et al., 2018; Lima et al., 2019). It is mainly caused by the key human pathogen *Aspergillus fumigatus*, a saprophytic filamentous fungus with widespread existence in the environment (Bertuzzi et al., 2014; Van De Veerdonk et al., 2017). Currently, several types of antifungal drugs, including azoles, polyenes, and echinocandins, are used for clinical aspergillosis treatment (Stiller et al., 1955; Maertens, 2004; Valiante et al., 2015). Azoles were deemed first-line oral treatment due to their higher efficacy and fewer side effects (Roemer and Krysan, 2014; Khalfe and Rosen, 2022), which are antifungals that act by binding to the target protein, Cyp51, a lanosterol 14α-demethylase, to inhibit the biosynthesis of the membrane component ergosterol (Li et al., 2019; Perez-Cantero et al., 2021). However, with an increase in the number of immunocompromised patients, such as those with AIDS, cancer, or organ transplants, the rising emergence of azole-resistant fungal pathogens resulting from the abuse of azoles in clinics and agriculture has been a new challenge for clinical aspergillosis treatment (Camps et al., 2012; Chowdhary et al., 2013; Verweij et al., 2016).

Regarding the mechanism of fungal pathogen resistance to azoles, previous studies have reported three main reasons: the mutation of the drug target Cyp51, overexpression of drug efflux pumps, and activation of cellular stress responses (Shapiro et al., 2011). Much of the current research on azole resistance is focused on the expression or modification of the drug target Cyp51 (Bueid et al., 2010; Perez-Cantero et al., 2020). Recent estimates for azole resistance displayed that more than 50% of the isolations included non-Cyp51 mutants that exhibit azole resistance for unexplained causes (Ener et al., 2022). Hence, additional treatment strategies are necessary for azole resistance to the human fungal pathogen *A. fumigatus*.

In eukaryotes, mitochondria are known as the powerhouse and are involved in a series of biological processes, such as Ca^2+^ homeostasis, apoptosis, and biosynthesis of lipids, nucleotides, amino acids, and other metabolites, by providing the raw materials for metabolism and the universal energy ATP (Jeong and Seol, 2008; Vakifahmetoglu-Norberg et al., 2017; Akbari et al., 2019; Rossi et al., 2019). Full functional mitochondria are a crucial guarantee for the orderly functioning of multiple cellular processes (Akbari et al., 2019). In the past decade, several studies have implied that mitochondria have a close-knit connection with fungal azole tolerance. In the yeast *Saccharomyces cerevisiae*, dysfunction of mitochondria by loss of the mitochondrial DNA (mtDNA) or deletion of mitochondrial membrane proteins causes a dramatic increase in azole resistance (Zhang and Moye-Rowley, 2001; Gohil et al., 2004). Moreover, mitochondria are highly dynamic organelles that continue to undergo fission and fusion to adjust to the different environmental conditions regulated by the conserved GTPase family of proteins Dnm1 and Fzo1, respectively (Okamoto and Shaw, 2005; Youle and Van Der Bliek, 2012). Fzo1 mutation results in mitochondrial fusion defects along with increased susceptibility to azole in *Candida albicans* (Thomas et al., 2013). By contrast, all three fission mutations (Δ*fis1*, Δ*mdv1*, and Δ*dnm1*) in *A. fumigatus* led to increased resistance to azole drugs (Neubauer et al., 2015). In *C. glabrata*, respiratory deficiency due to mutations in respiratory chain complexes results in decreased susceptibility to azoles (Peng et al., 2012). In summary, dysfunctional mitochondria resulting from distinctive reasons contribute to azole susceptibility in fungi. However, the mechanisms underpinning the relationship between mitochondrial and fungal response to azoles remain unclear.

Miro GTPase Gem1, conserved from fungi to mammals, is anchored to the mitochondrial outer membrane by the transmembrane domain at its C-terminus (Wolff et al., 1999; Fransson et al., 2003; Frederick et al., 2004). The remaining part is floating in the cytosol and senses the changes in Ca^2+^ concentration by its two EF-hand domains (Fransson et al., 2003). Gem1 is well-discovered in the budding yeast *S*. *cerevisiae*, where it contributes to maintaining mitochondrial morphology and mitochondrial inheritance, and facilitates the connection between mitochondria and other organelles by regulating the activity of the ER-mitochondria encounter structure (Frederick et al., 2004, 2008; Guillen-Samander et al., 2021). Moreover, the Gem1 homolog in the insect pathogen *Beauveria bassiana* is involved in stress response and virulence by affecting the mitochondrial function (Guan et al., 2016), and the Gem1 in *Neurospora crassa* showed a critical role in regulating the endoplasmic reticulum mitochondria encounter structure (ERMES) function (Wideman et al., 2013b). Importantly, the core components of ERMES complex, including Mmm1, Mdm10, Mdm12, and Mdm34, are well-discovered in *A. fumigatus* (Geissel et al., 2017), and they have gained interest to be used as drug targets, because no homologs exist in humans and animals (Wideman et al., 2013a). Furthermore, Gem1 has a crucial role in ensuring the mitochondrial morphology and cell wall stress response by regulating the MAPK pathway activity in *C. albicans* (Koch et al., 2017). However, the characteristics of Gem1 orthologs and their functions in mitochondria and antifungal drug response in *A. fumigatus* are still elusive. We here identified the yeast Gem1 homolog protein referred to as GemA in *A. fumigatus*, which is located in mitochondria and participates in maintaining the mitochondrial membrane potential (MMP). Further analysis indicated that GemA is required for hyphal growth, virulence, and cell wall integrity. Importantly, GemA exhibited a critical function in azole resistance by affecting the expression of a series of drug efflux pumps.

## Materials and methods

### Strains, media, and cultural conditions

All *A. fumigatus* strains used in this study are summarized in Table 1. In general, the strains were grown on a minimal medium (MM) or MMUU (MM supplemented with 5 mM uridine and 10 mM uracil) pH 6.5, containing 50 mL/L salt, 1% glucose, 1 mL/L 1,000 × trace elements, and 2% agar (Gupta et al., 1976). For all liquid media, agars were spared. The transformation was performed as per the protocol (Szewczyk et al., 2006), and hygromycin B (200 μg/mL, Shanghai Sangon Co., China) was used as a selection marker. All strains were cultured at 37°C for 1.5–3 days.

**Table 1:** *Aspergillus fumigatus* strains used in this study.

**Strains**	**Genotype**	**Reference or source**
A1160	*Δku80, pyrG*	FGSC
A1161	*Δku80, A1160::pyrG*	Jiang et al., 2014
XGA01	*Δku80, pyrG, ΔgemA::pyrG*	This study
XGA02	*Δku80, pyrG, ΔgemA::pyrG, gemA::gemA::hph*	This study
XGA03	*Δku80, pyrG, gemA::gfp::pyrG*	This study
XGA04	*Δku80, pyrG, gpd(p)-rfp-mrsA-hph*	This study
XGA05	*Δku80, pyrG, ΔgemA::pyrG, gpd(p)-rfp-mrsA-hph*	This study
XGA06	*Δku80, pyrG, gemA::gfp::pyrG, gpd(p)-rfp-mrsA-hph*	This study
XGA07	*Δku80, pyrG, Δmdr1::hph*	This study
XGA08	*Δku80, pyrG, ΔgemA::pyr4, Δmdr1::hph*	This study

### Construct design and protein tagging

All primers used for constructing deletion mutants, complementary strain, and protein tagging strains in this study are shown in Table 2. The previously described fusion PCR procedure was used to generate the *gemA* deletion cassette (Szewczyk et al., 2006). Briefly, ~1 kb of the upstream and downstream fragments of the open reading frame (ORF) of *gemA* was amplified using primers *gemA*-P1/P3 and *gemA*-P4/P6, respectively, and the genomic DNA (gDNA) of A1160 was used as a template. To gain a selectable marker, the *pyrG* fragment was amplified from the template plasmid pXDRFP4 by using primers pyrG-F/pyrG-R. Next, the deletion cassette was generated using primers *gemA*-P2/P5 and the aforementioned three fragments as templates. Subsequently, the PCR products were purified and then transformed into the recipient strain A1160. A similar strategy was used to generate *mdr1* deletion mutants. The aforementioned transformants were cultured in MM or MM plus hygromycin B when used with UU (uridine and uracil) or hygromycin B alone as selectable markers and verified through diagnostic PCR.

**Table 2:** Primers used in this study.

**Name**	**Sequence (5^′^-3^′^)**
gemA-P1	GCTGACTCCGGCAAATAGACC
gemA-p2	CCCTATCGACCGAGCAATTAGCTG
gemA-p3	CCTCTAGATGCATGCTCGAGCTGGTACTGGTTACGTTCTTCTAT
gemA-p4	CATCAGTGCCTCCTCTCAGACAGGACTTTGAGCTTATTAATCCC
gemA-p5	CTGTTCCTTGCTCGTCCCT
gemA-p6	TAAGCGACTGGCACGGTGTAG
gemA-F	ACTTAGCTGCCGATCATACCG
gemA-R	AGCCGAGATAAGCCAGATACTCC
PyrG-F	GCCTCAAACAATGCTCTTCACC
PyrG-R	CTGTCTGAGAGGAGGCCTGATG
C-gem-F	ACCTGCAGGCATGCAAGCTT TCATTGTACATGGTGTCTCCGTA
C-gem-R	CGACGGCCAGTGCCAAGCTTATTGGTTAGTCGATCACGCAT
gemA-gfp-p1	ACCTGAGCGACAAGGATCGAG
gemA-gfp-p2	TCCCCGATTCGAAGTACCA
gemA-gfp-p3	CCAGCGCCTGCACCAGCTCC TCCACTGCCGCTTACTCTTCG
gemA-gfp-p4	CATCAGTGCCTCCTCTCAGACAGTAGGACTTTGAGCTTATTAATCCCTACC
gemA-gfp-p5	TTCGCAGTTGTTATCCAGC
gemA-gfp-p6	GCCTTTGTGAAGAGAATCTTTGC
gfp-pyrG F	GGAGCTGGTGCAGGCGCTGG
gfp-pyrG R	CTGTCTGAGAGGAGGCACTGATG
Cla1-RFP-F	CCTTTAATCAAGCTTATCGATATGGCCTCCTCCGAGGACGTC
RFP-R	GGCGCCGGTGGAGTGGCGGCCC
RFP-Mrs-F	GGGCCGCCACTCCACCGGCGCCGCAGTCCCCGAGCAAATCCCAG
RFP-Mrs-R	CTCGAGGTCGACGGTATCGATTCACTCCTGGCGTTTGAAGTAGG
mdr1-P1	CGTACTCTGTACCTACCGACT
mdr1-p2	ATACCTGTAAAGGCAACTCGTC
mdr1-p3	GCACCGGTCAACCATGATCTGTTGCGGTTGAGGGTATGC
mdr1-p4	CACTCCACATCTCCACTCGATCATTCCTTCCCCCTTCTTCT
mdr1-p5	ACGATTACGAATGCCCGAT
mdr1-p6	CGACACGTCACGTACAATAGG
mdr1-F	AAGGCACGTACTATAAACTTGTGG
mdr1-R	CATTCTTTGCCTTACCCATGTCC
Hyg-F	AGATCATGGTTGACCGGTGC
Hyg-R	TCGAGTGGAGATGTGGAGTG
RT-tubA F	TTCCGTCCCGACAACTTCGT
RT-tubA R	TCACAGCCTTCAGCCTCACG
RT-tpo1A F	CTGCGCCCTATTCAAATGCTT
RT-tpo1A R	GAAAAGGTACAGGATTCCGTACACCA
RT-mdr1 F	GCTCTGCCTGAAGGTTACG
RT-mdr1 R	TGTGGGCTGTTTTGATTGT
RT-atrB F	CTGGCCTCGACGGTCAATCC
RT-atrB R	TTGGCCAACAGCAACAGGGT
RT-ste6A F	AACAAGAGATTGCGTTCTTCGAC
RT-ste6A R	ATACGCAATTCCCAAGACGGACA
RT-abcA F	CGGGCTTTTGGATTTTCATGTACC
RT-abcA R	TCAATATCTGAGCACTTGACGCTGG
RT-ste6B F	AAAACATCTCACAGGGGATGC
RT-ste6B R	GACACAAAGTCCCATGCGTT
RT-atrA F	GCATCCACGAGTCCAAGCGA
RT-atrA R	CCGCGCATATGCCAAGCATC
RT-tpo3 F	CCCCTTTATCCCGATCGCAA
RT-tpo3 R	CCGAACGCAGGAACACCTT
RT-tpo1B F	CGAGTATGCCGCCAAATCCG
RT-tpo1B R	CGCATCTGCCGAACATCCG
RT-abcD F	CAGAAGCAACGCATCGCCAT
RT-abcD R	CTCTTGGACAATAGCCTCCGACT
RT-vba1 F	GCCCCGCTTTAGTGACGAAT
RT-vba1 R	CCGCCCAAGATCAGTACCTT
RT-vba5 F	TGGTGCTTTCTGATCAACCTTCCG
RT-vba5 R	GAGCATGACTCCACTGCGAAT
RT-gel2 F	TAGCGGCGAGTCCAATACCC
RT-gel2 R	CCGCTGCACTCTCCTTGTCA
RT-eglB F	TCGACCAAGACCACCACTAGC
RT-eglB R	TACAGACACTGAGAGTACCACGGAT
RT-aspf2 F	CTGGCTTGTCTCCATGTGCTC
RT-aspf2 R	CCAGGGCAATCACCTCGTC
RT-chiC F	TTCTGAATCGTGCAGCGCAAC
RT-chiC R	CACAGCTAATAGTCAACGAGGT

To generate the *gemA* complementary strain, the full-length ORF of the *gemA* gene combined with its native promoter fragment (~1.5 kb upstream of *gemA* ORF) was amplified using primers c-gemA-F/c-gemA-R, with gDNA of A1160 as a template. The resulting PCR fragments were cloned into the plasmid pAN7-1, which carries a hygromycin B selectable marker. The cloned products were transformed into the background strain Δ*gemA*.

To generate a green fluorescent protein (GFP)-labeled GemA strain, the left fragment without the termination codon of *gemA* and the right fragment including the termination codon were amplified using the primers gemA-gfp-p1/gemA-gfp-p3 and gemA-gfp-p4/ gemA-gfp-p6, with A1160 gDNA as a template, respectively. The GFP-pyrG fragment, including a fluorescence GFP tag and a selectable maker *pyrG*, was amplified using the primers GFP-pyrG-F/GFP-pyrG-R and the template plasmid pFN03. Next, the aforementioned three PCR products were used as templates to generate the fusion cassette by using primers gemA-gfp-p2/gemA-gfp-p5. The resulting fragments were purified and transformed into the background strain A1160 to obtain the GFP-labeled strain, referred to GemA-GFP.

For generating the red fluorescent protein (RFP)-tagged MrsA strain, the ORF of *mrsA* (including the termination codon) was amplified from the A1160 gDNA by using the primers mrsA-F/mrsA-R. The *rfp* fragment (except for the termination codon) was amplified using the primers RFP-F/RFP-R, with the plasmid pXDRFP as a template. Next, the aforementioned *rfp* fragment and the *mrsA* fragment were combined using the primers RFP-F/mrsA-R. The resulting fusion fragments were then cloned into the pBARGPE1 vector (carrying an *AngpdA* promoter) (Song et al., 2016), which then were transformed into the background strain A1160, Δ*gemA*, and GemA-GFP, respectively.

### Microscopy and image processing

To visualize the localization of proteins, including GemA and MrsA, conidia of the indicated strains were inoculated onto sterile glass coverslips and incubated for 16 h at 37°C. Before the detection, the cells were rinsed three times with phosphate buffer saline (PBS) and then fixed with 4% paraformaldehyde. Images were captured using an Olympus BX53 microscope (Tokyo, Japan) and managed with ImageJ [ImageJ (nih.gov)] and Adobe Photoshop (Adobe, San Jose, CA, USA).

### *G. mellonella* virulence assay

Virulence observations in *G. mellonella* were performed as described (Fallon et al., 2011). In general, 20 larvae as a group, 1 × 10^5^ conidia (dissolved in 10 μL PBS) of the indicated strains were injected into the larvae and 10 μL PBS was used as a control. Next, the injected larvae were incubated at 37°C. Then, the mortality was recorded daily and the survival rate was analyzed using GraphPad Prism software with Kaplan–Meier survival curves.

### RNA sequencing analysis, RNA isolation, and RT-qPCR

For RNA sequencing (RNA-seq) analysis, 1 × 10^8^ conidia of the *gemA* deletion mutant and its parental wild-type (WT) strains were incubated in liquid MM for 18 h at 37°C. After incubation, mycelium was filtered and immediately harvested in liquid nitrogen. The samples were then sent to Personal Biotechnology (Nanjing, China) for the following sequencing. The raw sequence reads have been submitted to SRA (https://www.ncbi.nlm.nih.gov/sra) at NCBI with six accession numbers SRR19440562–SRR19440564 (WT-1, WT-2, and WT-3, three biological replicate samples of WT) and SRR19440565–SRR19440567 (ΔgemA-1, ΔgemA-2, and ΔgemA-3, three biological replicate samples of Δ*gemA*).

For RNA isolation, the sample preparation is similar to that described in RNA-seq analysis. The total RNA was obtained using the purification kit (UNIQ-10 column, Sangon). Digestion and reverse transcription were performed using the HiScriptII Q RT SuperMix for the qPCR (gDNA wiper) kit from Vazyme to obtain cDNA. qPCR was then performed using the AceQ qPCR SYBR green master mix (Vazyme). All procedures were performed according to the manufacturer's instructions.

### MMP analysis

The experiments were performed to detect the MMP in the related strains as described previously (Wei et al., 2017). In summary, 2.5 × 10^6^ fresh conidia were cultured in YG medium at 37°C until they germinated. Then, the samples were then washed with PBS and incubated with a final concentration of 10 μM rhodamine 123 (Rh123, Beyotime) for 30 min at 37°C. Next, extracellular Rh123 was gently removed and washed three times with PBS. The fluorescence intensity was determined using flow cytometry (FACS, BD FACSVerse).

## Results

### Identification of the yeast Gem1p homolog in *A. fumigatus*

**Figure 1:**
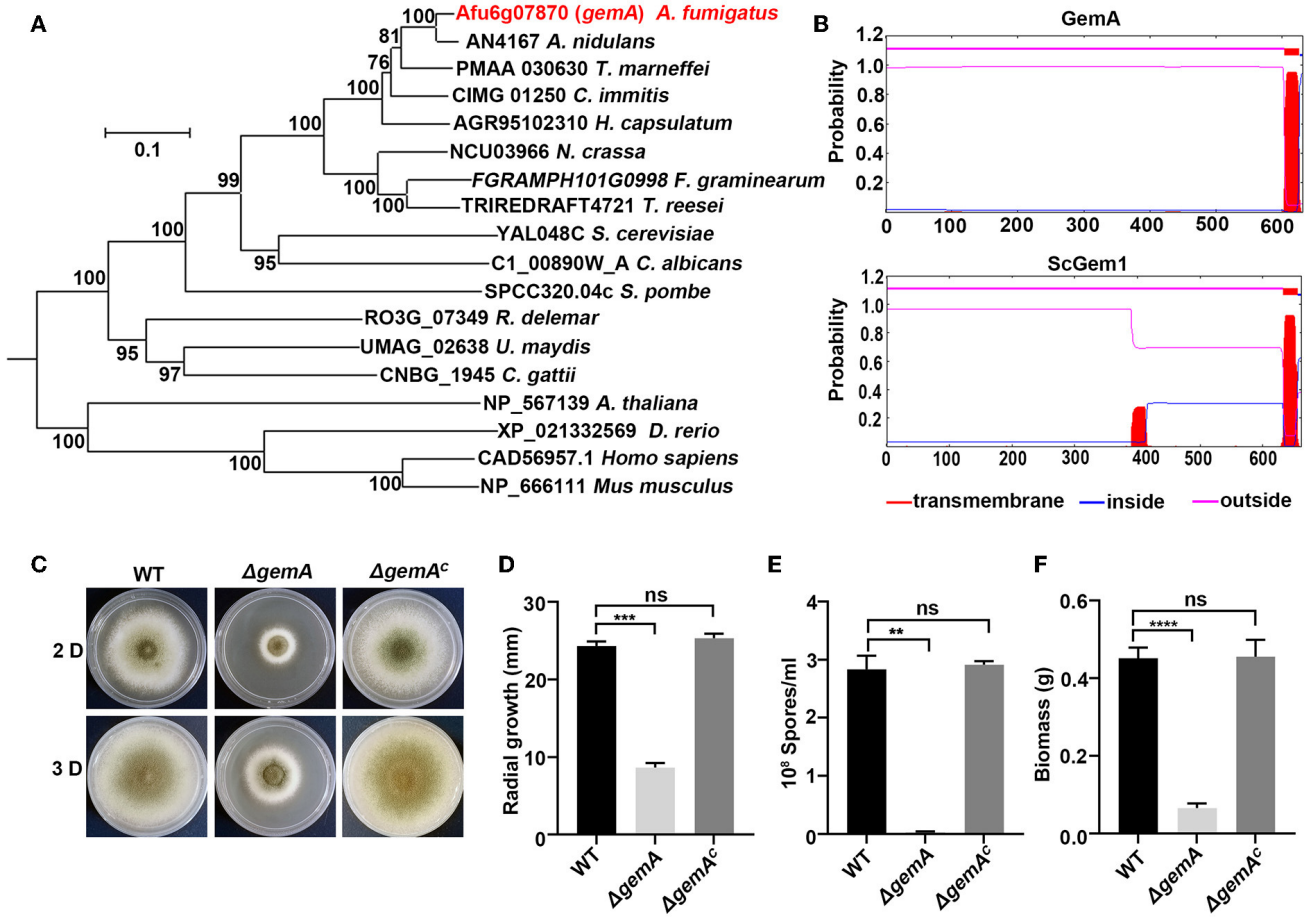
Bioinformatic analysis of GemA and phenotypic characterization of the *gemA* mutant. **(A)** Phylogenetic analysis of GemA homologs from selected species. The MEGA 6 software was used to generate the phylogenetic tree. **(B)** The transmembrane domain prediction of GemA in *A. fumigatus* and ScGem1 in *S. cerevisiae* by using the TMHMM server v. 2.0 (http://www.cbs.dtu.dk/services/TMHMM-2.0). **(C)** Phenotypes of the indicated strains cultured on MM at 37°C for 2 or 3 days, and their quantitative data for **(D)** colony size **(E)**, conidia production **(F)**, and biomass. ns, *P* > 0.05; **P < 0.01; ***P < 0.001; ****P < 0.0001.

To identify the putative Gem1p (GTPase EF-hand protein of mitochondria) homologs of budding yeast, *S. cerevisiae*, in *A. fumigatus*, the amino acid sequence of ScGem1 was used as a query to perform BLASTP analysis in the genome database of *A. fumigatus*. The search yielded a putative mitochondrial GTPase Afu6g07870 (XP_750678.1, identity 49%, *E*-value 0.0). Subsequently, BLASTP analysis in the *S. cerevisiae* genome database by using Afu6g07870 as a query showed ScGem1 as the best match. The results suggested that Afu6g07870 was a possible ScGem1 homolog in *A. fumigatus*, which was referred to as GemA. A phylogenetic relationship analysis revealed that Gem-related proteins are conserved from the filamentous fungi to mammals (Figure 1A). The deduced *gemA* DNA sequence is 1,959-bp long and encodes a protein with 632 amino acids. Moreover, the conserved domain analysis using the TMHMM v2.0 or A Simple Modular Architecture Research Tool (SMART) program showed that both GemA and ScGem1 contain a transmembrane structure at their C-terminus, two GTPase domains, and two EF-hand (a calcium-binding domain) domains (Figure 1B and Supplementary Figure 1A).

### Lack of GemA results in decreased hyphal growth in *A. fumigatus*

To further investigate GemA functions in *A. fumigatus*, a deletion mutant strain Δ*gemA* was constructed through homologous integration in the background of the WT strain A1160. A single null mutant of Δ*gemA* was verified through diagnostic PCR (Supplementary Figure 1B), indicating that the *gemA* ORF was successfully deleted. As shown in Figures 1C–F, the *gemA* deletion mutant exhibited a series of defective phenotypes (e.g., an attenuated colony diameter, impaired conidiation, and reduced biomass) compared with the phenotypes of its parental WT strain. By contrast, the *gemA* complementary strain (verifying through diagnostic PCR, as shown in Supplementary Figure 1C) displayed no detectable difference from the WT strain (Figures 1C–F), suggesting the phenotypes could be rescued by reintroducing the *gemA* gene into the mutant. These data also indicated that GemA has a crucial role in hyphal growth in *A. fumigatus*.

### GemA localizes mitochondria in *A. fumigatus*

**Figure 2:**
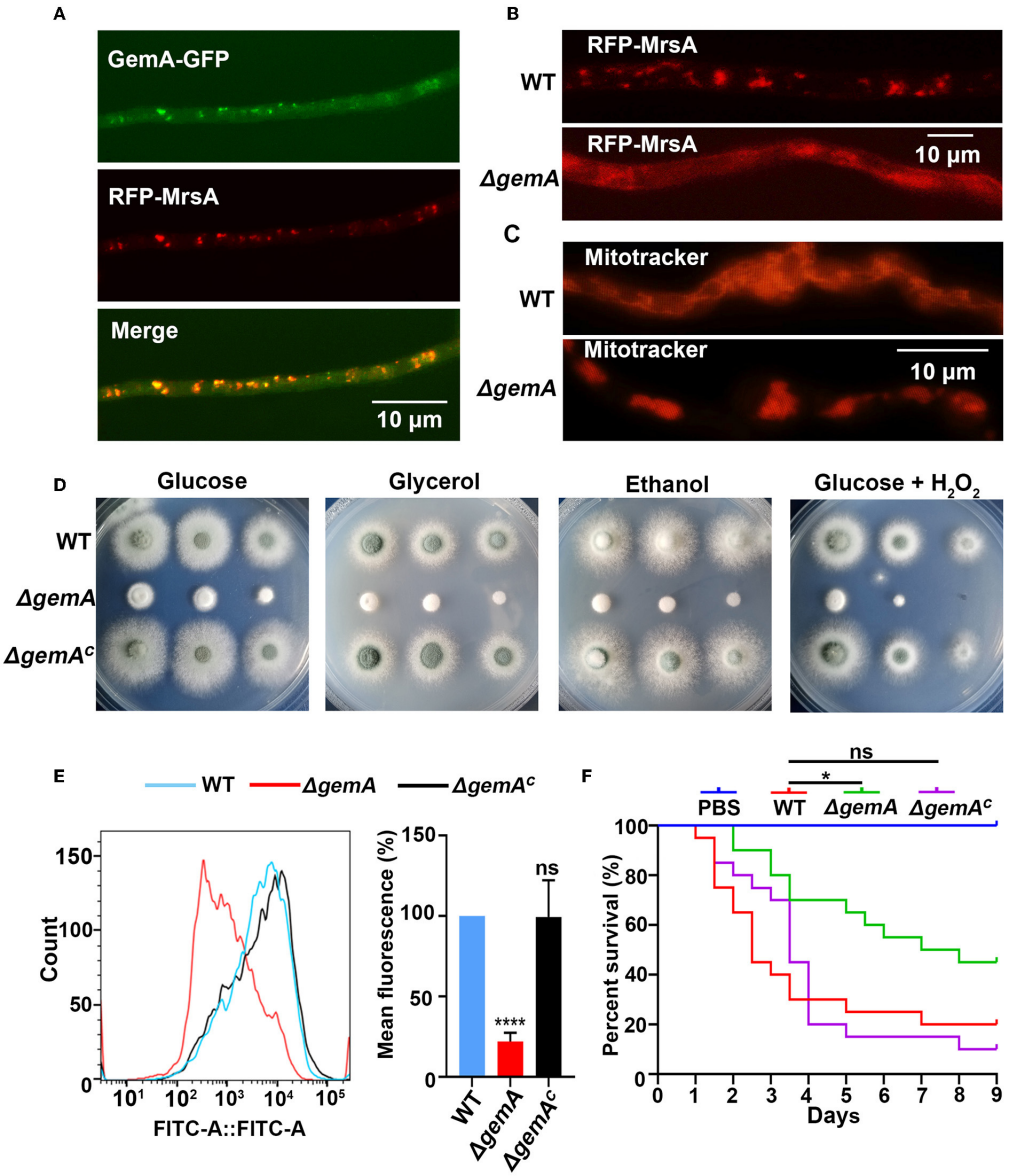
Loss of GemA disrupted the mitochondrial function in *A. fumigatus*. **(A)** Subcellular localization of GFP-tagged GemA and **(B)** RFP-MrsA in the indicated strains. Bars, 10 μm. **(C)** The mitochondrial morphological characteristics of the indicated strains. Mitochondria were stained by MitoTracker red. Bars 10 μm. **(D)** Colony morphologies of the wild-type (WT) strain, Δ*gemA*, and *gemA* complementary strain cultured in MM containing different carbon sources (1% glucose, 1% glycerol, or 1% ethanol) or the oxidation reagents H_2_O_2_ (3 mM) at 37°C for 2 days. **(E)** The mitochondrial membrane potential was determined through flow cytometry. The fluorescence intensity value is shown on the x-axis. **(F)** Survival curve of *G. mellonella* infected with WT, Δ*gemA*, Δ*gemA*^*C*^, and PBS. ns, *P* > 0.05; *P < 0.05; ****P < 0.0001.

To gain further insights into GemA, an *in situ* GFP-labeled strain was constructed through homologous integration in which the GFP was tagged to the C-terminus of *gemA* under the control of its native promoter (Supplementary Figure 2A). As shown in Supplementary Figure 2A, colony phenotypes of the GemA-GFP strain were similar to those of its parental WT strain, indicating that the GFP-tagged GemA protein was functional. Fluorescence microscopy revealed that the GemA-GFP protein had a mitochondria-like localization pattern (Figure 2A and Supplementary Figure 2B), consistent with ScGem1 in the budding yeast (Frederick et al., 2004). To directly verify whether GemA is located in mitochondria, we performed an experiment labeling the mitochondrial marker MrsA (Long et al., 2016) with the RFP to its N-terminus under an *AngpdA(p)* promoter in the background of strain GemA-GFP. Subsequent microscopy revealed that the red fluorescence pattern well-overlapped the GFP fluorescence signal, suggesting GemA localizes to mitochondria in *A. fumigatus* (Figure 2A and Supplementary Figure 2B).

### GemA is required for mitochondrial morphology, MMP, and virulence in *A. fumigatus*

The aforementioned data showed that GemA is a mitochondrial protein in *A. fumigatus*. Therefore, we evaluated whether GemA is necessary for the proper functioning of mitochondria. For this, two fluorescent strains were constructed, in which mitochondria were visualized by labeling the mitochondrial marker MrsA with the RFP tag in the background of the *gemA* deletion mutant and the WT strain. Microscopic observation revealed that the localization of RFP-MrsA is more diffused in the *gemA* deletion mutant than in the WT reference strain (Figure 2B and Supplementary Figure 2C). To further investigate whether GemA plays a role in maintaining mitochondrial morphology, the mitochondrial-specific dye, Mito-Tracker Red CMXRos, was used to visualize the mitochondria in the strains of WT and Δ*gemA*. Fluorescence observation revealed that filiform or tubular mitochondria were present in the WT strain, while an aberrant giant mitochondrial morphology was observable in the *gemA* deletion mutant strain (Figure 2C and Supplementary Figure 2D), suggesting GemA is required for the proper localization of MrsA to mitochondria and the maintaining of mitochondrial morphology.

Mitochondria are essential organelles for stress response and energy metabolism. Hence, to further explore whether the loss of GemA impacts the mitochondrial function, the Δ*gemA* mutants, complementary strain, and WT strain were cultured in media supplemented with non-fermentative carbon sources, such as glycerol or ethanol and the oxidation reagent H_2_O_2_. As shown in Figure 2D and Supplementary Figure 2E, except for the growth defect result from the mutation of *gemA*, no significant difference was exhibited between the Δ*gemA* mutants and the WT or complementary strains in those adverse conditions. Previous studies reported that the respiratory complex I activity is crucial for the mitochondrial function (Bromley et al., 2016; Kroll et al., 2016); then, the activity examination assays were carried out in the strain of WT and *gemA* deletion mutant. As shown in Supplementary Figure 2F, the mitochondrial complex I activity is unaffected in *gemA* deletion mutant. To provide further insights into the functions of GemA in mitochondria, the MMP of *gemA*-related strains was detected using a fluorescent MMP indicator, Rh123, through flow cytometry. Consequently, the *gemA* mutant displayed a significantly decreased MMP compared with its parental WT strain, which suggested that GemA is actually participating in maintaining the MMP in *A. fumigatus* (Figure 2E).

The *gemA* null deletion mutant severely impaired colony growth and mitochondrial function. Whether GemA contributed to virulence in *A. fumigatus* remains unclear. The *gemA*-related strains were tested in a *G. mellonella* model of invasive aspergillosis. The survival of *G. mellonella* was monitored for 10 days at 37°C after infection. As shown in the survival curves of Figure 2F, the Δ*gemA* mutant exhibited a significantly reduced mortality rate (*P* < 0.01) of larvae compared to its parental WT strain, whereas the *gemA* complementary strain revealed a similar mortality rate as the WT strain, indicating virulence was attenuated in the Δ*gemA* mutant. Taken together, the aforementioned data suggested that GemA is required for colony growth, conidiation, proper mitochondrial functions, and virulence in *A. fumigatus*.

### GemA is involved in resistance to azoles and terbinafine, but not amphotericin B

**Figure 3:**
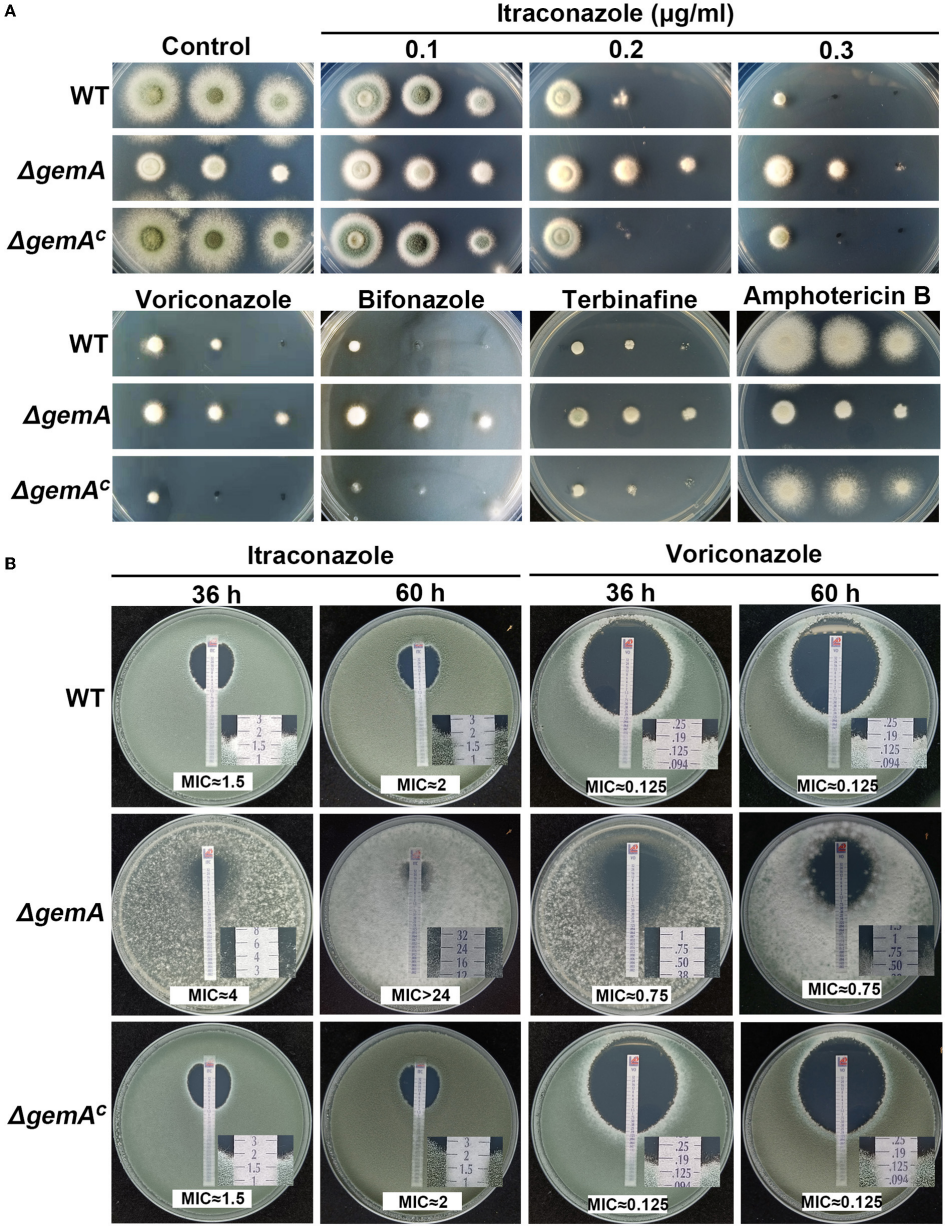
Loss of GemA increased resistance to azoles. **(A)** Colony morphologies of the indicated strains cultured in MM supplemented with itraconazole (ITC; 0.1, 0.2, and 0.3 μg/mL), voriconazole (0.3 μg/mL), bifonazole (0.7 μg/mL), terbinafine (0.6 μg/mL), and amphotericin B (6 μg/mL) at 37°C for 2 days. **(B)** The MIC test of the related strains was conducted using the commercialized *E*-test strip. Conidia (1 × 10^5^) of the WT or Δ*gemA* were mixed in YAG, and then, the ITC or voriconazole test strip was placed on the plate and incubated at 37°C for 36 h or 60 h.

Many lines of evidence have indicated that mitochondria participate in numerous biological processes through energy production, including resistance to antifungal drugs (Thomas et al., 2013; Long et al., 2016; Song et al., 2020). Hence, we speculate GemA may be involved in drug responses. To verify this hypothesis, the Δ*gemA* mutants, complementary strain, and WT strain were cultured in media supplemented with different concentrations of the antifungal drug itraconazole (ITC). As shown in Figure 3A, the Δ*gemA* mutants exhibited heightened antifungal resistance to ITC compared with its parental WT strain regardless of the drug concentration. By contrast, no difference in colony phenotypes was observed between the *gemA* complementary strain and WT strain when grown in the presence of ITC, indicating that the phenotype of increased resistance to ITC truly results from loss of GemA. To further investigate whether GemA contributes to resistance to other antifungal drugs, we conducted drug susceptibility assays by using other antifungal drugs, including azoles (voriconazole and bifonazole), allylamines (terbinafine), and polyenes (amphotericin B). As shown in Figure 3A, by comparison, the Δ*gemA* mutants showed more robust than the WT strain in the presence of azoles and allylamines, but not in the presence of polyenes. We next tested the MIC values of GemA-related strains by using commercial E-test strips. As shown in Figure 3B, the MIC value of ITC for the Δ*gemA* mutant (≈4 μg/mL at 36 h and >24 μg/mL at 60 h) was significantly higher than that of the WT strain and the *gemA* complementary strain (≈1.5 μg/mL at 36 h and ≈2 μg/mL at 60 h). Similarly, by using voriconazole E-test strips, the Δ*gemA* mutant (≈0.75 μg/mL) showed a higher MIC value than the reference WT (≈0.125 μg/mL) and *gemA* complementary strains (≈0.125 μg/mL). This MIC test further demonstrated that GemA is involved in antifungal azole response in *A. fumigatus*. Considering GemA as GTPase located in the mitochondrial membrane, they may indirectly influence the azole response by affecting the other genes' expression.

### GemA mutation induces upregulation of a series of azole resistance-related transport genes

Abnormal biosynthesis of the cell membrane component, ergosterol, has been recognized as the principal cause of fungal resistance to azoles. RT-qPCR revealed that the key genes of the ergosterol biosynthesis pathway in the Δ*gemA* mutant, including the azole target *cyp51*, were normally expressed compared with those in the WT strain (Supplementary Figure 3A).

**Figure 4:**
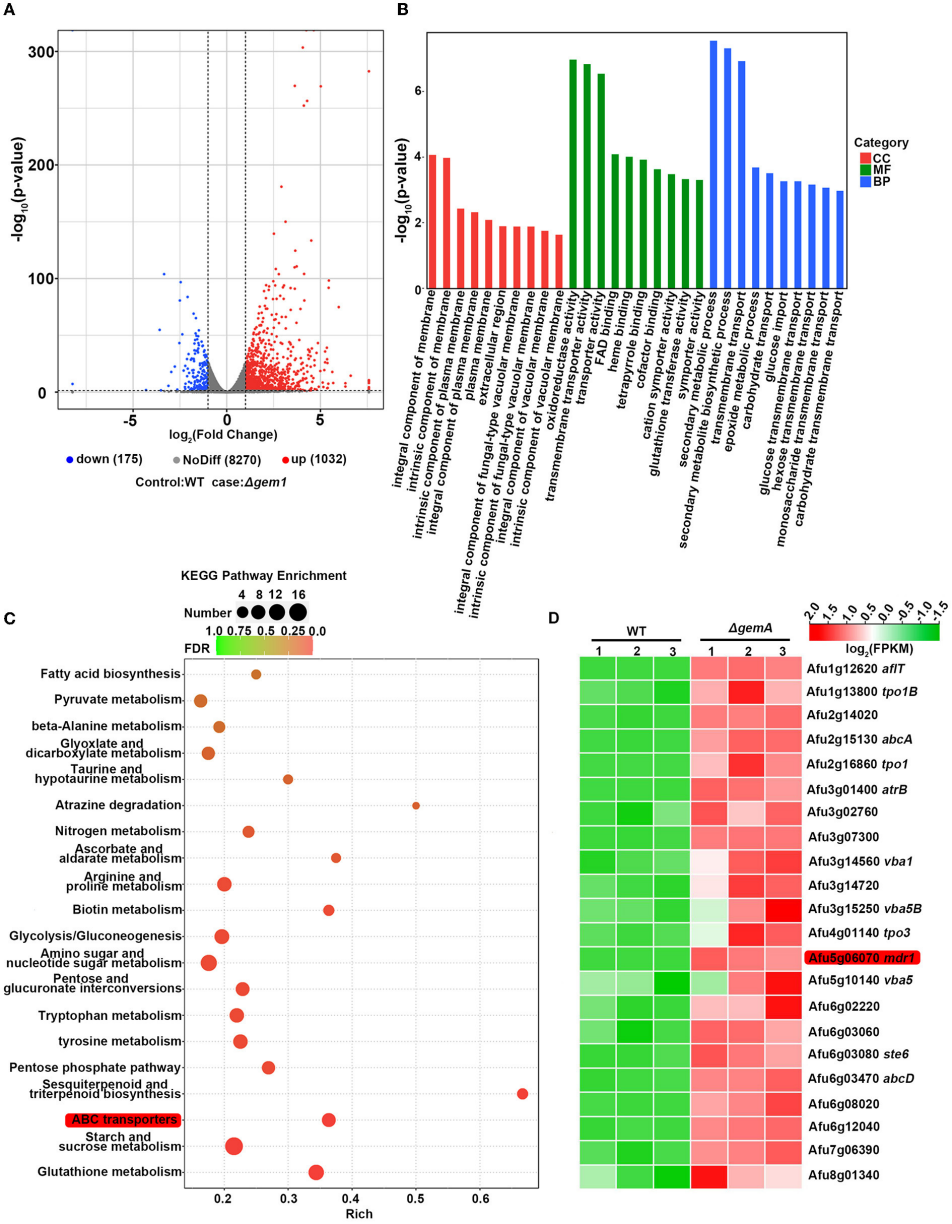
RNA-seq data analysis in the *gemA* mutant and its parental WT strain. **(A)** The volcano plot showed the differentially expressed genes in the *gemA* mutant compared with the WT strain. **(B)** Functional category and **(C)** signal pathway enrichment analyses were performed using GO and KEGG databases. **(D)** A heatmap comparison of drug efflux pump-related genes between the *gemA* mutant and its parental WT strain by using RNA-seq data.

To further investigate the molecular mechanism underlying drug resistance in GemA-related mutants, we performed RNA-seq to analyze the overall transcriptional profiling of the azole-resistant strain Δ*gemA* and its parental WT strain (a control). The total read number of each sample is shown in Supplementary Table 1, and the reads map to the genome database by the software HISAT2 (http://ccb.jhu.edu/software/hisat2/index.shtml; Supplementary Table 2). Next, the expression level was normalized with FPKM (fragments per kilobase per million fragments). The volcano analysis indicated a series of differentially expressed genes, including 175 downregulation genes and 1,032 upregulation genes (adjusted *P*-value ≤ 0.05, log2FC ≥ 1) (Figure 4A) (Dataset 1). Furthermore, the GO category analysis exhibited that the amounts of proteins involved in transmembrane transport were enriched (Figure 4B). Moreover, the subsequent signal pathway enrichment analysis performed using the Kyoto Encyclopedia of Genes and Genomes (KEGG) database showed that the aforementioned differentially expressed genes were mainly mapped to metabolic processes. Of them, ATP-binding cassette (ABC) transporters (Figure 4C), a type of fungal drug efflux pumps (Sturm et al., 2020), were enriched, suggesting that *gemA* deletion may be affected by intracellular drug residues in the fungus. In addition, as shown in Figure 4D, the selected genes, including those encoding the ABC transporters and major facilitators superfamily (MFS, another type of drug efflux pump), were broadly upregulated in the Δ*gemA* mutant compared with the WT strain. Notably, the well-known drug efflux pump *mdr1* was significantly upregulated (25-fold).

### Loss of *mdr1* attenuates the degree of azole resistance that results from the mutation of GemA

**Figure 5:**
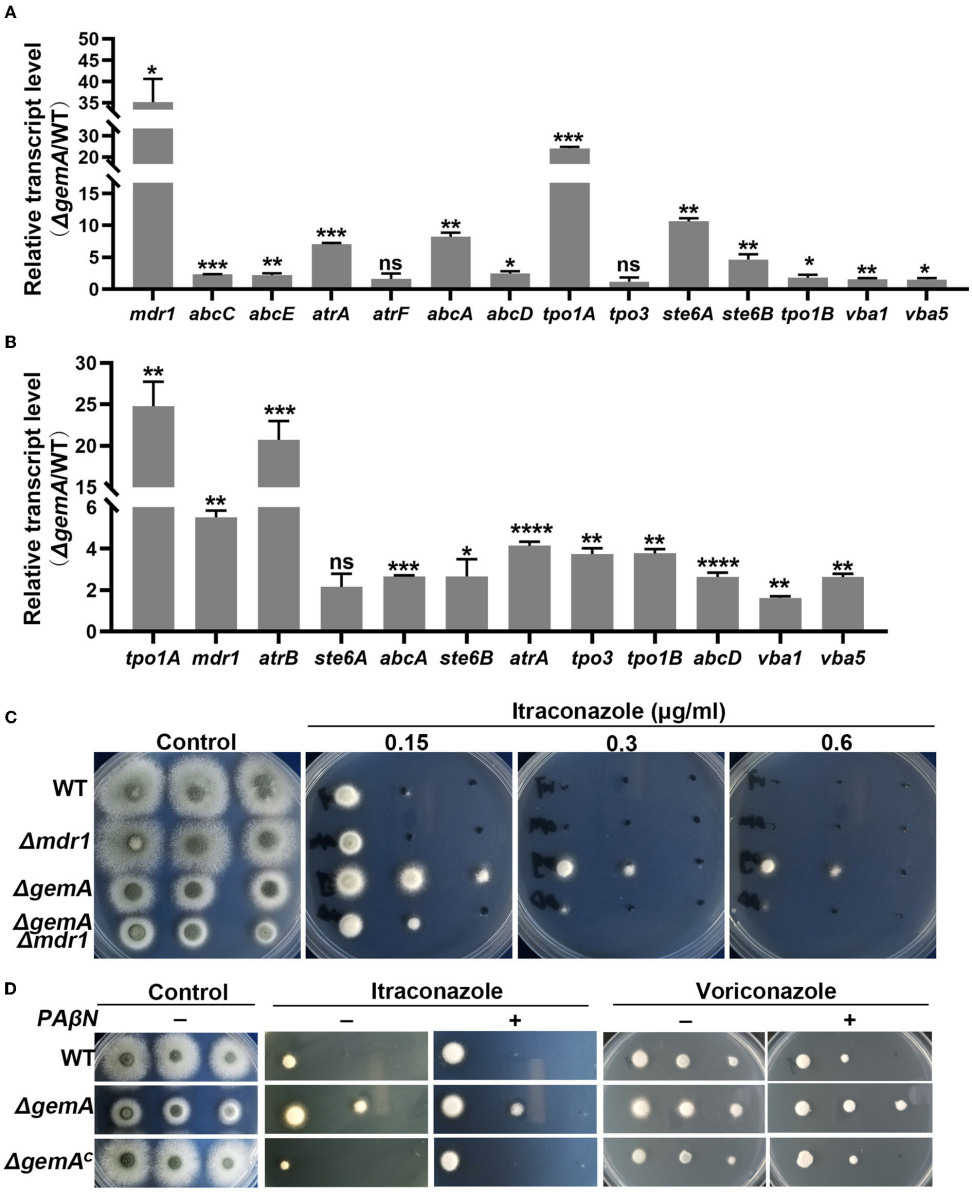
GemA affected the expression of drug efflux pump-related genes. A quantitative RT-PCR comparison of indicated genes between the *gemA* mutant and its parental WT strain **(A)** under cultured in MM media at 37°C for 24 h or **(B)** transforming to 0.1 M ITC for 30 min. **(C)** Colony morphologies of the *gemA* mutant, *mdr1* mutant, double mutant *gemA* /*mdr1*, and WT strain (a control) cultured in MM with or without the antifungal drug ITC at 37°C for 2 days. **(D)** The colony morphology of the indicated strains on MM with or without various reagents, including drugs (0.2 μg/mL ITC or 0.15 μg/mL voriconazole) and a pan-efflux inhibitor *PA*β*N* (64 μg/mL). ns, *P* > 0.05; *P < 0.05; **P < 0.01; ***P < 0.001; ****P < 0.0001.

To verify the aforementioned results of RNA-seq, quantitative real-time PCR (qRT-PCR) was performed using the biological replicate samples. As shown in Figure 5A, the expression levels of the majority of the selected genes increased to different degrees in the *gemA* deletion mutant compared with the WT strain, which was consistent with the results of RNA-seq. Considering the overexpression of efflux pumps is meaningful for fungi during drug exposure, we then examine efflux pump expression levels in the strains of WT and Δ*gemA* after stimulation with 0.1 M ITC in 30 min. As shown in Figure 5B, the majority of selected efflux pumps were increased expression in the Δ*gemA* compared to its parental WT strain. To further test whether the decreased azole susceptibility phenotype in the Δ*gemA* strain results from the increased expression of the aforementioned drug efflux pumps, the key drug resistance-related gene *mdr1* was deleted in the background of the *gemA* deletion mutant and the WT strain. As shown in Figure 5C, the loss of *mdr1* largely rescued azole susceptibility in the Δ*gemA* mutant when grown in the presence of ITC, indicating that GemA contributes to azole susceptibility by affecting the expression of drug efflux pumps. In addition, the susceptibility to itraconazole (but not voriconazole) was slightly rescued in the Δ*gemA* strain when cultured in a pan-efflux inhibitor phenylalanine-arginine beta-naphthylamide (*PA*β*N*) media (Figure 5D; Rajendran et al., 2011; Lu et al., 2018), implying that azole susceptibility in the *gemA* deletion mutant is related to drug efflux in *A. fumigatus*.

A recent study showed that dysfunctional mitochondria increased the expression of drug efflux pumps by perturbing cellular calcium homeostasis to activate the Ca^2+^-dependent CrzA signaling pathway (Li et al., 2020), combined with the two Ca^2+^-binding domains (EF hands) present in GemA (Supplementary Figure 1A). We wonder whether azole resistance in the Δ*gemA* mutant is linked to the CrzA signaling pathway. As shown in Supplementary Figure 3B, the Δ*gemA* mutant displayed no significant difference in the colony phenotype whether cultured in the medium supplemented with EDTA (a calcium chelating) or CaCl_2_. Moreover, the Δ*gemA* mutant showed resistance to azoles even when the CrzA signaling pathway was disrupted by FK506 (Supplementary Figure 3B), an anti-calcineurin agent, suggesting the overexpression of drug efflux pumps may not be caused by the calcium signal.

### GemA is required for cell wall composition and integrity

**Figure 6:**
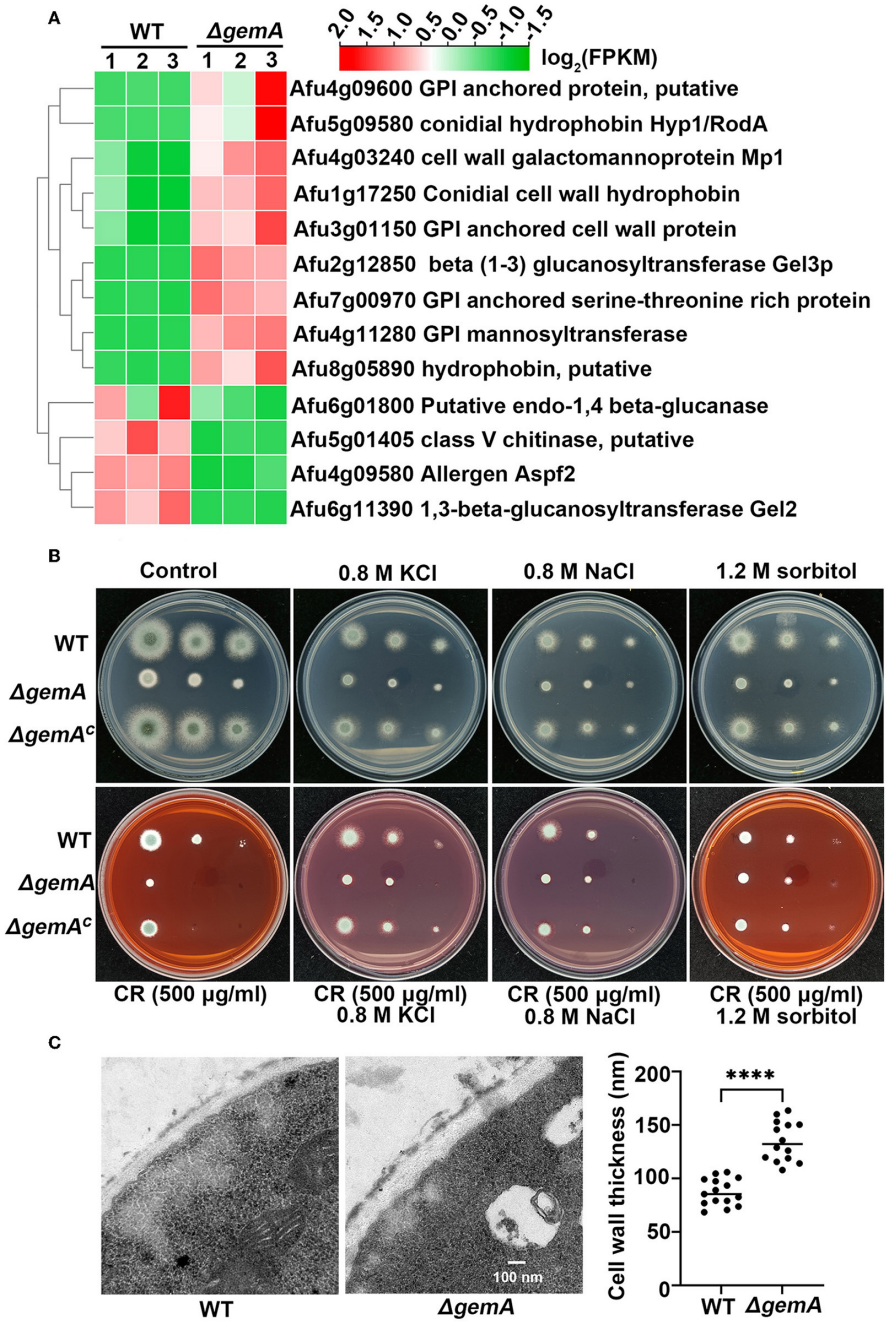
GemA is required for response to cell wall stress in *A. fumigatus*. **(A)** A heatmap comparison of cell wall-related genes between the *gemA* mutant and its parental WT strain by using RNA-seq data. **(B)** A colony phenotype comparison of the indicated strains in MM with or without a cell wall-perturbing reagent Congo red and hyperosmotic stress regents (KCl, NaCl, and sorbitol) at 37°C for 2 days. **(C)** The transmission electron microscopy images and quantitative data of cell wall thickness of the *gemA* mutant and its parental WT strain. ****P < 0.0001.

RNA-seq analysis demonstrated that a series of cell wall-related genes, such as those encoding GPI-anchored protein, cell wall hydrophobin, chitinase, and glucanase, were differentially expressed in the *gemA* deletion mutant compared with the parental WT strain (Figure 6A). The following qRT-PCR showed a transcriptional profile similar to the results of RNA-seq (Supplementary Figure 4A), indicating that GemA may play a role in cell wall integrity in *A. fumigatus*. To verify this hypothesis, GemA-related strains were cultured in the presence of the cell wall-perturbing agents Congo red (CR) and the chitin-binding dye calcofluor white (CFW). As shown in Figure 6B and Supplementary Figure 4B, the Δ*gemA* mutant showed more sensitive to CR, but not to CFW, than its parental WT strain. By comparison, the *gemA* complementary strain showed a normal phenotype when grown under CR stress, implying loss of *gemA* results in a cell wall defect. Interestingly, this defective phenotype under CR stress was partially rescued through supply with the hyperosmotic stress reagents, including NaCl, KCl, and sorbitol, while hyperosmotic stress cannot improve the colony growth of the *gemA* mutant in the normal MM media (Figure 6B). Moreover, to explore the role of GemA in cell wall architecture, the cell wall thickness of the *gemA* deletion mutant and WT strain was observed through transmission electron microscopy (TEM). Notably, the cell wall thickness of the *gemA* deletion strain was 2-fold thicker than that of its parental strain (Figure 6C). Taken together, our data suggested that GemA is required for the maintenance of cell wall integrity and composition in *A. fumigatus*.

## Discussion

### GemA is a mitochondrial protein in *A. fumigatus*

In many human pathogenic fungi, strategies of dysfunctional organelles are compromised to overcome numerous environmental stresses (e.g., azole resistance; Li et al., 2020). An increasing number of studies have corroborated that the mitochondria as dynamic organelles play important roles in fungal resistance to azoles (Okamoto and Shaw, 2005; Peng et al., 2012; Youle and Van Der Bliek, 2012; Neubauer et al., 2015), although the mechanism is still not understood. This study identified GemA, a homolog of Miro GTPase Gem1 in the budding yeast *S. cerevisiae*, that regulates mitochondrial function in the filamentous pathogenic fungi *A. fumigatus*. Several pieces of evidence support GemA as a mitochondrial protein. First, GemA is the homolog of the Miro GTPase Gem1 in yeast, as observed through homologous comparison (Figure 1A). Moreover, domain analysis revealed that consistent with Gem1 in yeast, GemA had two Miro GTPase domains, two EF hands, and a transmembrane domain at its C-terminus (Figure 1B and Supplementary Figure 1A). Second, the GFP-tagged GemA covered the mitochondrial marker MrsA-RFP, which showed a mitochondria-like localization pattern (Figure 2A and Supplementary Figure 2B). Third, the GemA mutant showed an abnormal mitochondrial morphology compared with its parental WT strain (Figure 2C and Supplementary Figure 2D). Finally, GemA was required to maintain an appropriate MMP (Figure 2E). Our results demonstrated that GemA plays a critical role in appropriate mitochondrial function in *A. fumigatus* and was evolutionarily conservative in fungi.

### GemA is required for virulence

Our study also found that deletion of the mitochondrial protein GemA results in reduced virulence in *A. fumigatus* (Figure 2F), which agrees with its homolog in *B. bassiana* (Guan et al., 2016). Pathogens that cause the disease need to fit in the host environment and competitively utilize the host's nutrients to combat macrophage phagocytosis (Heinekamp et al., 2015). The *gemA* deletion strain showed a sicker colony phenotype and reduced biomass compared with the WT strain (Figures 1C–F), suggesting that the fitness of the Δ*gemA* strain was reduced by dysfunctional mitochondria. This may be the reason for crippled virulence in the *gemA* deletion mutant. Moreover, a previous study reported that mitochondria with a tubular morphology and with more efficient mitochondrial functions contribute to pathogenic virulence (Frederick et al., 2004). For example, the hypervirulence strain of *Cryptococcus gattii* was generated by changing the mitochondrial morphology to tubular. Interestingly, an aberrant giant mitochondrial morphology was observed in the GemA deletion mutant (Figure 2C and Supplementary Figure 2D), indicating that mitochondria with abnormal morphology and an inefficient function may be another cause of attenuated virulence in the *gemA* deletion mutant.

### GemA contributes to fungal susceptibility to azoles

At present, several lines of evidence have implied that mitochondrial function affects the susceptibility of human pathogen fungi to many antifungal reagents (Youle and Van Der Bliek, 2012; Thomas et al., 2013; Li et al., 2020). The data presented in this study demonstrated that GemA as a mitochondrial protein influences the response to azoles. Therefore, the *gemA* deletion mutant showed more resistance to azoles (including ITC, voriconazole, and bifonazole) than the WT strain, which is used as a control (Figure 3).

Studies have reported that abnormal biosynthesis of the cell membrane component ergosterol is the main reason for fungal resistance to azoles in the past decades (Alcazar-Fuoli and Mellado, 2012; Perez-Cantero et al., 2021). To identify the mechanism underlying the increased resistance of the *gemA* mutant strain to azoles, we first detected the mRNA transcriptional level of key enzymes in the ergosterol biosynthesis pathway, including that of the drug target *cyp51A* and *cyp51B*. Unexpectedly, the expression of none of them was different from that in the WT strain (Supplementary Figure 3A), suggesting that ergosterol biosynthesis may not change in the Δ*gemA* mutant. This deduction was also indirectly supported by colony morphology, as observed in Figure 3A, such that no detectable difference was observed between the Δ*gemA* mutant and WT strain cultured in a medium supplemented with amphotericin B, an antifungal drug that acts by binding the membrane component ergosterol. RNA-seq and qRT-PCR demonstrated that numerous drug efflux pumps were upregulated in the Δ*gemA* strain (Figures 4C,D, 5A,B). In addition, confirmatory experiments conducted by deleting the key drug efflux pump *mdr1* or supplying the pan-efflux inhibitor *PA*β*N* indicated that inhibiting the expression of drug efflux pumps can partly reduce azole resistance (Figures 5C,D). Altogether, our data in this study indicated that GemA contributed to azole susceptibility may be by affecting the expression of drug efflux pumps.

A recent study in *A. fumigatus* revealed a mechanism for azole resistance caused by the upregulated drug efflux pumps in strains with dysfunctional mitochondria due to an activated Ca^2+^-dependent CrzA signaling pathway triggered by the disturbed intracellular calcium concentration (Li et al., 2020). Another molecular mechanism proposed was shown in *C. glabrata* and *S. cerevisiae* wherein defective membrane lipid homeostasis in strains with dysfunctional mitochondria leads to the activation of the drug resistance pathway (Hallstrom et al., 2001; Batova et al., 2008). Our data showed no detectable difference in the *gemA* deletion mutant under low or high calcium culture with the supply of CaCl_2_ or EDTA (a cationic chelator) (Supplementary Figure 3B), although GemA has two EF-hand domains. In addition, the azole resistance phenotype in the *gemA* deletion mutant was not altered when the CrzA signaling pathway was disrupted using the immunosuppressor FK506 to inhibit the serine/threonine protein phosphatase, calcineurin (Supplementary Figure 3B). The aforementioned data suggested that the overexpression of drug efflux pumps in the Δ*gemA* strain may not be caused by the calcium signaling pathway. Numerous studies demonstrated that the mitochondria and its ERMES complex play an important role in maintaining the membrane lipid homeostasis (Wideman et al., 2013b; Geissel et al., 2017; Koch and Traven, 2020; Tamura et al., 2020), and Gem1 homolog, as a GTPase, regulated the ERMES function, such that loss of the *gemA* may disrupt the lipid homeostasis in the cellular membrane system. Previous studies suggested that abnormal membrane lipid homeostasis creates a signal to increase the efflux pump expression by activating the transcription factor, such as *Sc*Pdr3 in *S. cerevisiae* and *Cg*Pdr1 in *C. glabrata* (Shingu-Vazquez and Traven, 2011). Combing with our RNA-seq analysis by using the GO database that membrane component-related genes were influenced, suggesting that GemA may play a role in the membrane lipid homeostasis and membrane stability (Figure 4B), hence, we speculate that the mechanism of azole resistance in the *gemA* deletion mutant may involve membrane lipid homeostasis, but not the calcium signaling pathway. Taken together, the mitochondrial protein GemA influences the azole susceptibility by affecting the membrane lipid homeostasis in *A. fumigatus*.

### GemA is necessary for cell wall composition

Previous studies have implied that mitochondrial function is closely associated with cell wall integrity in *C. parapsilosis, C. albicans*, and *S. cerevisiae* (Koch et al., 2017; Koch and Traven, 2020). In this study, we further characterized the Δ*gemA* mutant. This mutant showed sensitivity to the cell wall-perturbing agent CR (but not to CFW) and was partly rescued by hyperosmotic stress (including NaCl, KCl, and sorbitol; Figure 6B and Supplementary Figure 4B), suggesting the cell wall had become defective due to the loss of GemA. Moreover, RNA-seq and RT-qPCR showed that both up- and downregulated cell wall-related genes were involved, and TEM data showed that the cell wall was thicker in the Δ*gemA* mutant than in its parental WT strain (Figure 6C). Collectively, our data implied that GemA plays pivotal functions in cell wall composition and homeostasis.

A recent study in *A. fumigatus* showed that mitochondrial dysfunction increased the expression of cell wall-related genes by triggering the Ca^2+^-dependent CrzA signaling pathway (Li et al., 2020). However, our data showed that no significant difference was detected in the *gemA* deletion mutant under low or high calcium culture conditions (Supplementary Figure 3B), implying that calcium may not be the main reason for defective cell wall in Δ*gemA* mutant. In *C. albicans*, Gem1 participates in cell wall integrity by regulating the conserved MAPK signal pathway (Koch et al., 2017), a known stress response pathway (cell wall stress is included) in the eukaryotic cell (Martinez-Soto and Ruiz-Herrera, 2017). Our data showed that defective colony growth under CR stress of Δ*gemA* mutant was partially rescued through supply with the hyperosmotic stress (Figure 6B), suggesting the requirement of GemA for cell wall integrity may affect the MAPK pathway. Further, the ERMES complex is regulated by GemA, which affords an important role in phospholipid trafficking between the ER and the mitochondria (Shingu-Vazquez and Traven, 2011). Abnormal phospholipid homeostasis could affect the activity of cell wall synthesis enzymes, the synthesis of the GPI anchor, and the activation of cell wall stress responding pathways (Shingu-Vazquez and Traven, 2011). Hence, the GemA-ERMES-phospholipid-cell wall geometric network may be the other reason for GemA influencing the cell wall integrity in *A. fumigatus*.

## Data availability statement

The datasets presented in this study can be found in online repositories. The names of the repository/repositories and accession number(s) can be found in the article/Supplementary material.

## Author contributions

XZ and XC: conception and design of the investigation and work. XZ, GY, and CL: completion of the experiments. XZ, GY, and FY: evaluation and analysis of the results. XZ: manuscript writing. XZ, GY, CL, FY, and XC: final approval of manuscript. All authors contributed to the article and approved the submitted version.

## Funding

This work was financially supported by the Natural Science Foundation of Anhui Province (Grant 2108085QC90), the National Natural Science Foundation of China (Grant 32100152), and the Scientific Research Foundation of the Higher Education Institutions of Anhui Province (Grant KJ2021A0775) to XZ. The Graduate Research and Innovation Projects of Bengbu Medical College (Grant Byycx21011) to GY.

## Conflict of interest

The authors declare that the research was conducted in the absence of any commercial or financial relationships that could be construed as a potential conflict of interest.

## Publisher's note

All claims expressed in this article are solely those of the authors and do not necessarily represent those of their affiliated organizations, or those of the publisher, the editors and the reviewers. Any product that may be evaluated in this article, or claim that may be made by its manufacturer, is not guaranteed or endorsed by the publisher.

## Supplementary material

Supplementary Figure 1 **(A)** Domain analysis of GemA in *A. fumigatus* and ScGem1 in *S. cerevisiae* by using SMART. **(B)** The diagnostic PCR of the *gemA* mutant in comparison with the parental WT strain and **(C)** the diagnostic PCR of the complementation of the gemA deletion mutant.

Supplementary Figure 2 GemA is located in mitochondria in *A. fumitagus*. **(A)** Colony morphologies of the WT and GFP-labeled GemA strains cultured in MM at 37°C for 2 days and the diagram illustrating the strategy for the construction of the GemA-GFP strain under the control of a native promoter. **(B)** Subcellular localization of GFP-tagged GemA. **(C)** RFP-MrsA in the indicated strains. Bars, 10 μm. **(D)** The mitochondrial morphological characteristics of the indicated strains. Mitochondria were stained by MitoTracker red. Bars 10 μm. **(E)** Quantitative data of the normalized colony diameter for the WT (1161), Δ*gemA*, and Δ*gemA*^*C*^ strains cultured in MM supplemented with the indicated carbon source at 37°C for 2 days. **(F)** Quantitative data of the mitochondrial complex I activity in the strain of WT and *gemA* mutant.

Supplementary Figure 3 **(A)** Quantitative RT-PCR comparison of the ergosterol biosynthesis-related genes between the *gemA* mutant and its parental WT strain. **(B)** Colony phenotype and the normalized colony diameter for the WT (1161), Δ*gemA*, and Δ*gemA*^*C*^ strains cultured in MM supplemented with indicated reagents at 37°C for 2 days.

Supplementary Figure 4 **(A)** Quantitative RT-PCR comparison of cell wall-related genes between the *gemA* mutant and its parental WT strain. **(B)** The colony phenotype of the WT (1161), Δ*gemA*, and Δ*gemA*^*C*^ strains cultured in MM supplemented with the CFW at 37°C for 2 days.

Supplementary Table 1 Disembarking data information.

Supplementary Table 2 RNA-Seq map information.
