# Supplementary figures and images for: Requirement of a putative mitochondrial GTPase, GemA, for azole susceptibility, virulence, and cell wall integrity in Aspergillus fumigatus
Source: Front Microbiol. 2022 Aug 9;13:957857. doi: 10.3389/fmicb.2022.957857 (PMC9396130; doi:10.3389/fmicb.2022.957857)

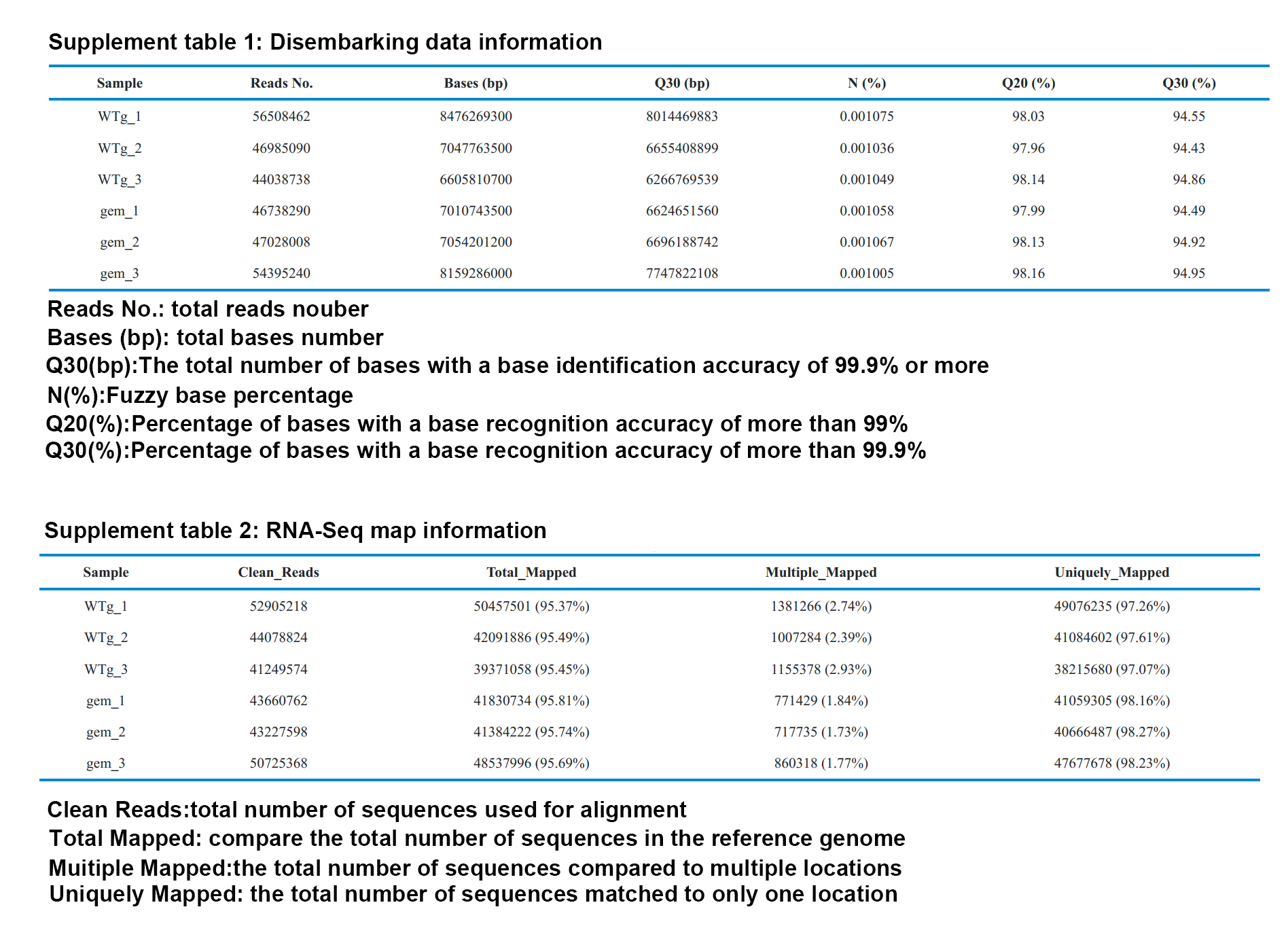

Supplement: Supplementary file 1 [file Image_5.JPEG]

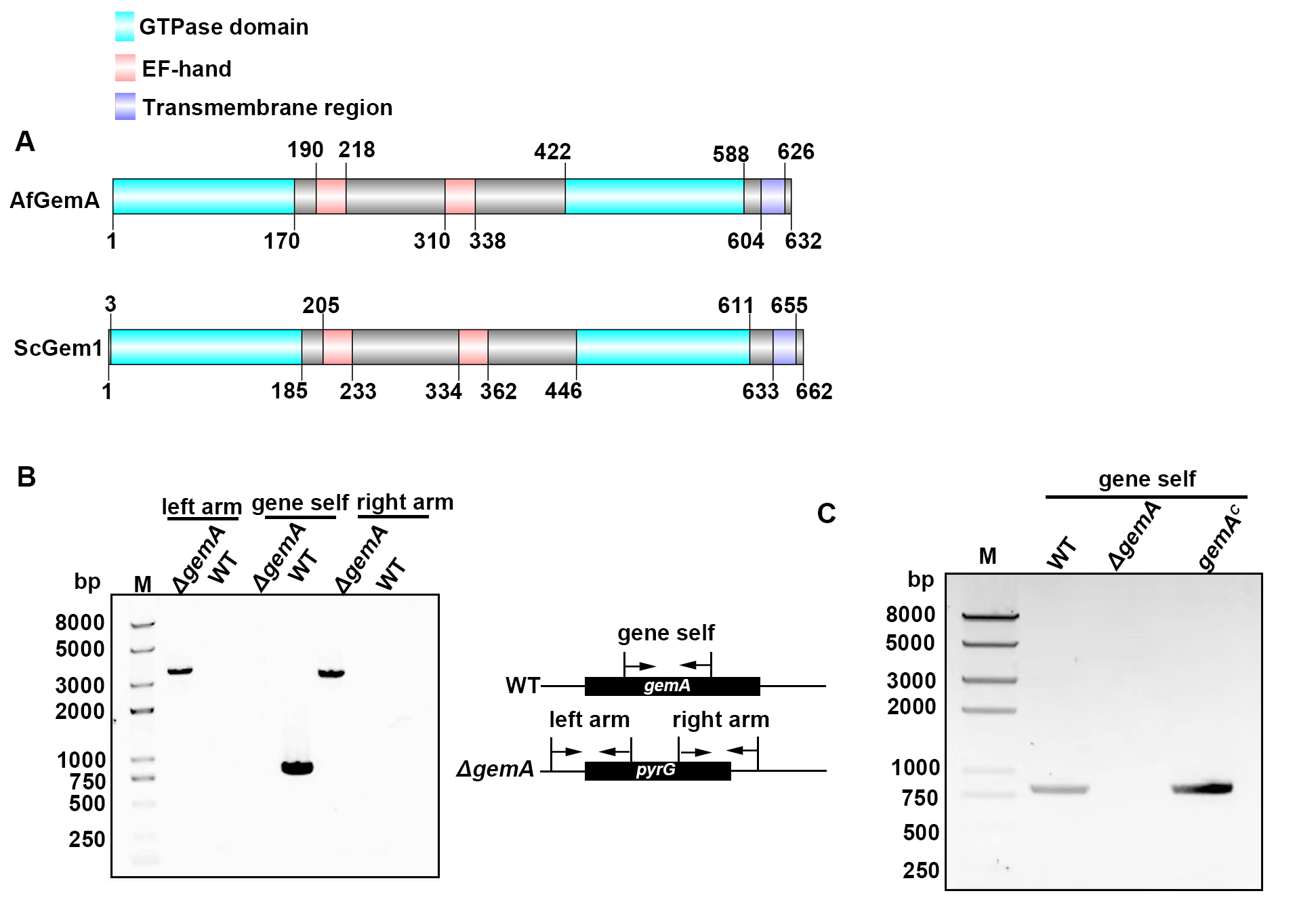

Supplement: Supplementary Figure 1 — (A) Domain analysis of GemA in A. fumigatus and ScGem1 in S. cerevisiae by using SMART. (B) The diagnostic PCR of the gemA mutant in comparison with the parental WT strain and (C) the diagnostic PCR of the complementation of the gemA deletion mutant. [file Image_1.JPEG]

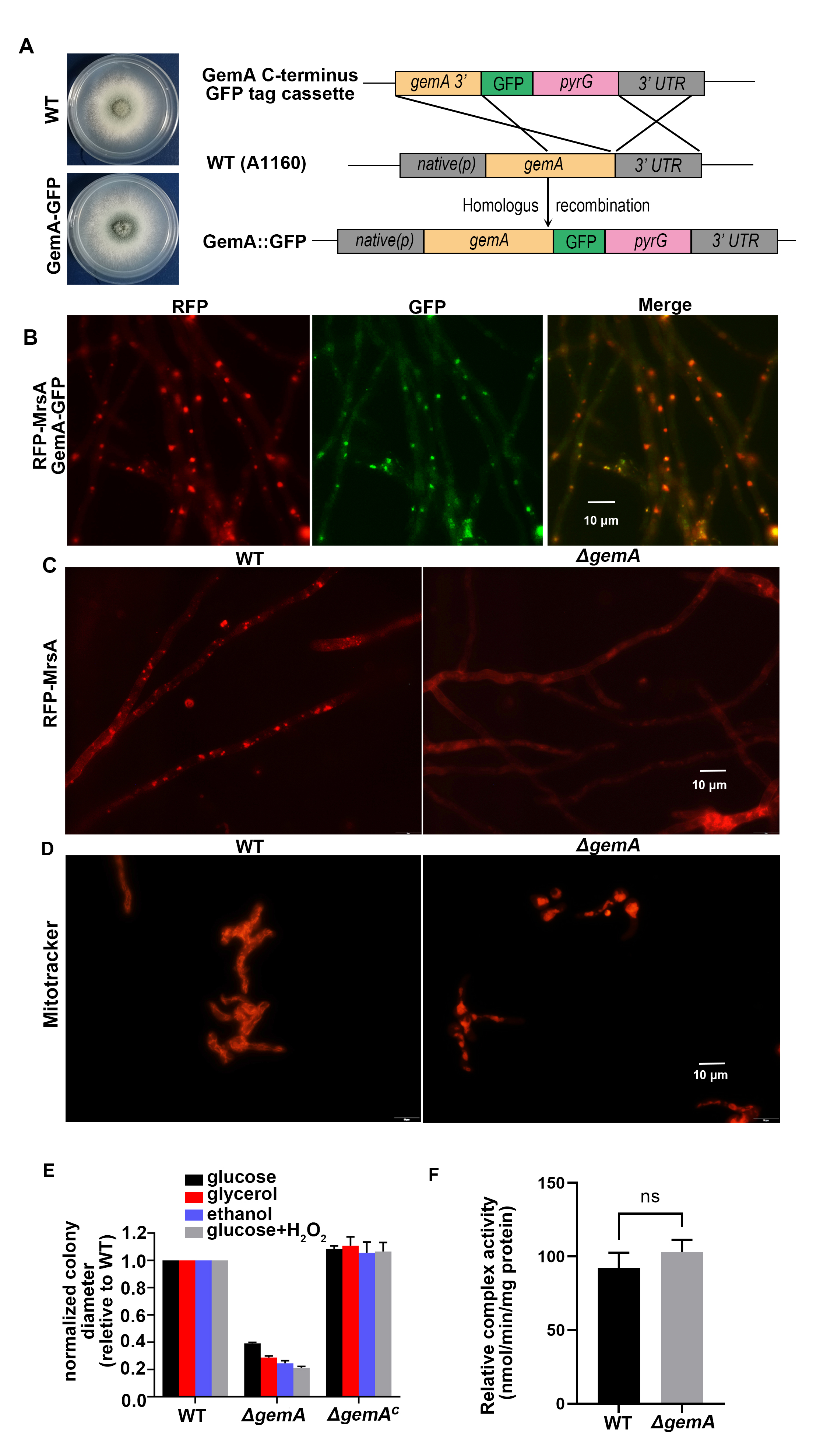

Supplement: Supplementary Figure 2 — GemA is located in mitochondria in A. fumitagus. (A) Colony morphologies of the WT and GFP-labeled GemA strains cultured in MM at 37°C for 2 days and the diagram illustrating the strategy for the construction of the GemA-GFP strain under the control of a native promoter. (B) Subcellular localization of GFP-tagged GemA. (C) RFP-MrsA in the indicated strains. Bars, 10 μm. (D) The mitochondrial morphological characteristics of the indicated strains. Mitochondria were stained by MitoTracker red. Bars 10 μm. (E) Quantitative data of the normalized colony diameter for the WT (1161), ΔgemA, and ΔgemAC strains cultured in MM supplemented with the indicated carbon source at 37°C for 2 days. (F) Quantitative data of the mitochondrial complex I activity in the strain of WT and gemA mutant. [file Image_2.JPEG]

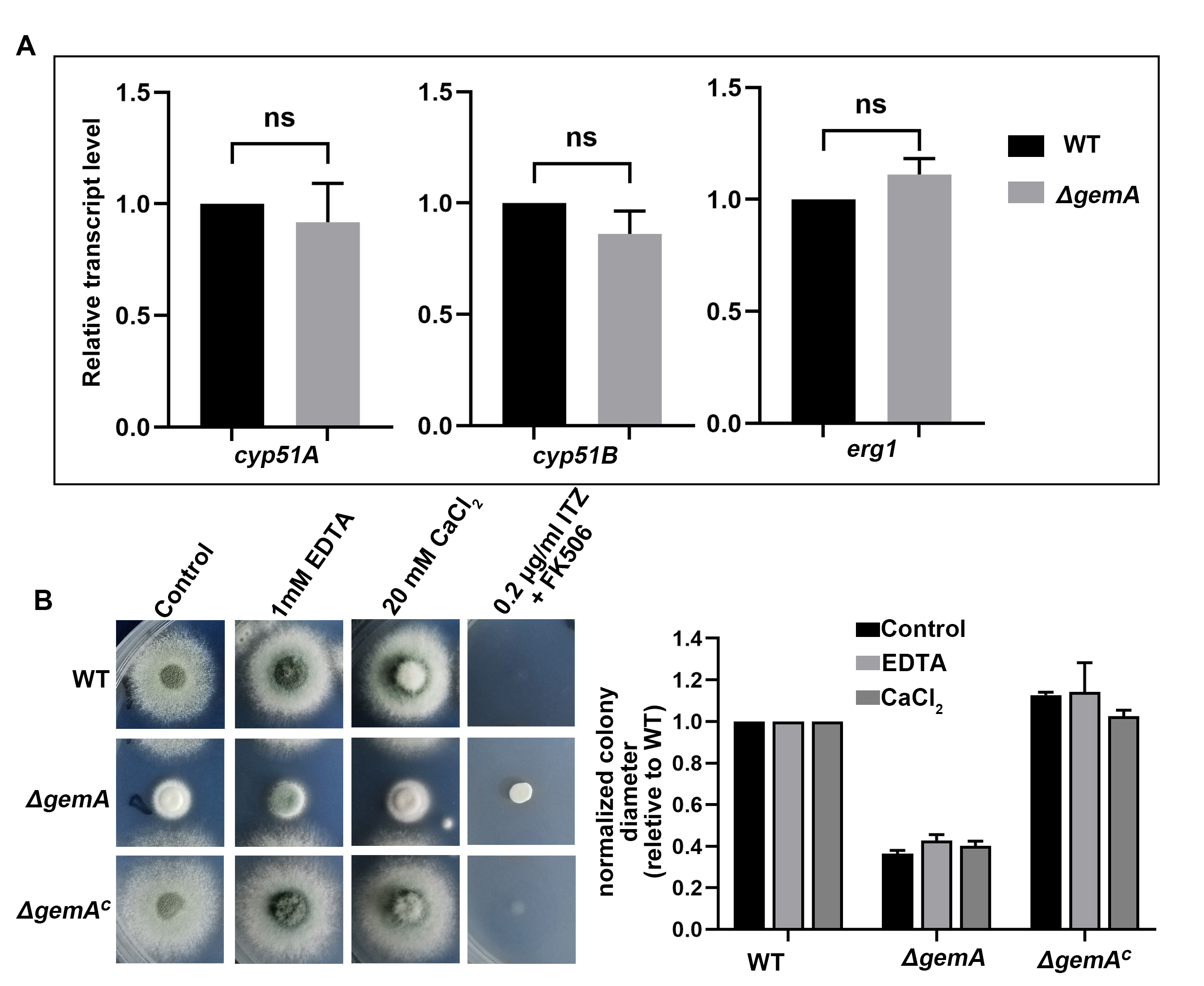

Supplement: Supplementary Figure 3 — (A) Quantitative RT-PCR comparison of the ergosterol biosynthesis-related genes between the gemA mutant and its parental WT strain. (B) Colony phenotype and the normalized colony diameter for the WT (1161), ΔgemA, and ΔgemAC strains cultured in MM supplemented with indicated reagents at 37°C for 2 days. [file Image_3.JPEG]

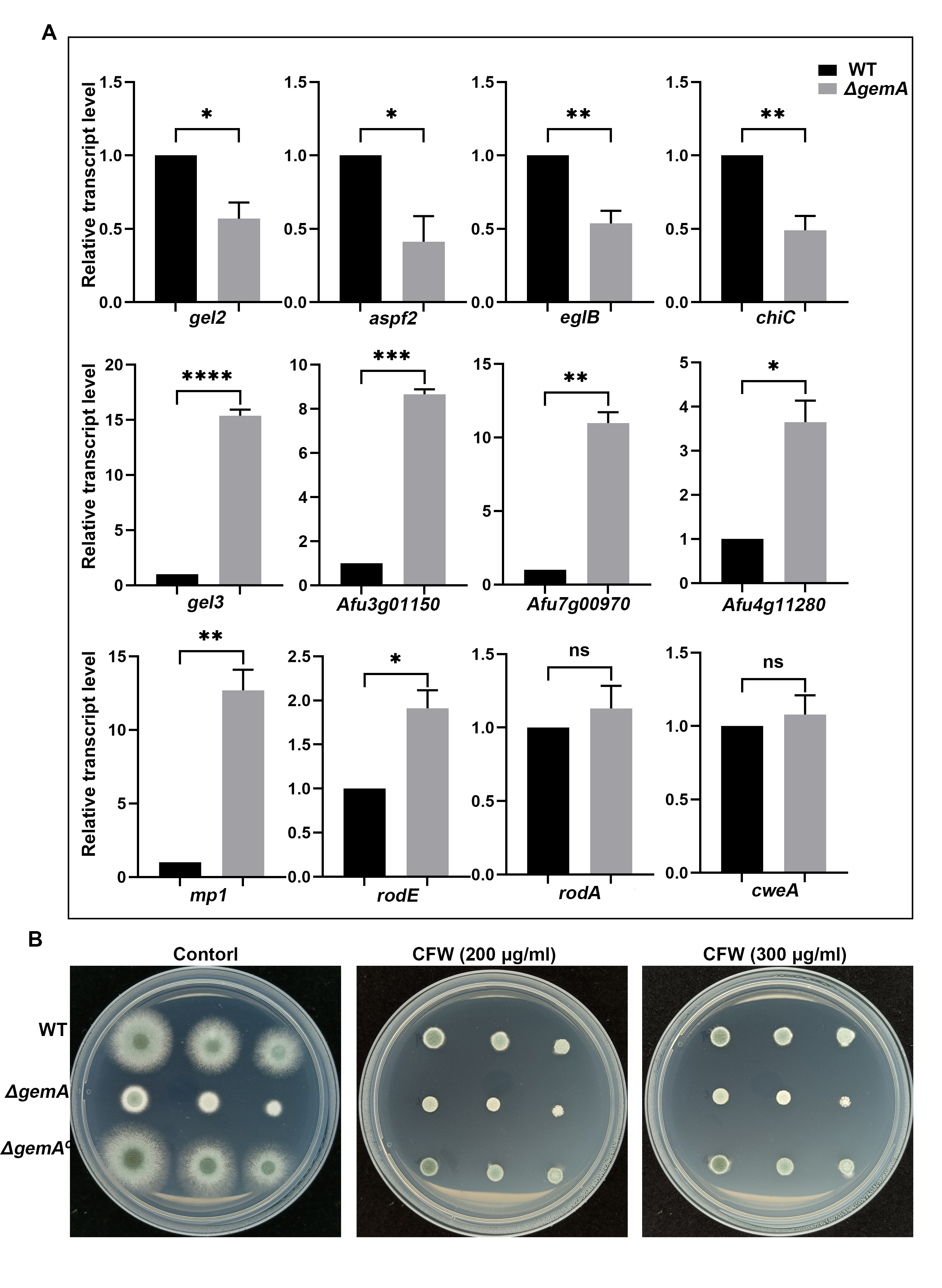

Supplement: Supplementary Figure 4 — (A) Quantitative RT-PCR comparison of cell wall-related genes between the gemA mutant and its parental WT strain. (B) The colony phenotype of the WT (1161), ΔgemA, and ΔgemAC strains cultured in MM supplemented with the CFW at 37°C for 2 days. [file Image_4.JPEG]
